# Advantages of multi-arm non-randomised sequentially allocated cohort designs for Phase II oncology trials

**DOI:** 10.1038/s41416-021-01613-5

**Published:** 2021-11-08

**Authors:** Helen Mossop, Michael J. Grayling, Ferdia A. Gallagher, Sarah J. Welsh, Grant D. Stewart, James M. S. Wason

**Affiliations:** 1 Population Health Sciences Institute, Newcastle University, Newcastle upon Tyne, UK; 2 Department of Radiology, University of Cambridge, Cambridge, UK; 3 Department of Oncology, Cambridge University Hospitals NHS Foundation Trust, Cambridge, UK; 4 Department of Surgery, University of Cambridge, Cambridge Biomedical Campus, Cambridge, UK

**Keywords:** Adaptive clinical trial, Randomized controlled trials

## Abstract

**Background:**

Efficient trial designs are required to prioritise promising drugs within Phase II trials. Adaptive designs are examples of such designs, but their efficiency is reduced if there is a delay in assessing patient responses to treatment.

**Methods:**

Motivated by the WIRE trial in renal cell carcinoma (NCT03741426), we compare three trial approaches to testing multiple treatment arms: (1) single-arm trials in sequence with interim analyses; (2) a parallel multi-arm multi-stage trial and (3) the design used in WIRE, which we call the Multi-Arm Sequential Trial with Efficient Recruitment (MASTER) design. The MASTER design recruits patients to one arm at a time, pausing recruitment to an arm when it has recruited the required number for an interim analysis. We conduct a simulation study to compare how long the three different trial designs take to evaluate a number of new treatment arms.

**Results:**

The parallel multi-arm multi-stage and the MASTER design are much more efficient than separate trials. The MASTER design provides extra efficiency when there is endpoint delay, or recruitment is very quick.

**Conclusions:**

We recommend the MASTER design as an efficient way of testing multiple promising cancer treatments in non-comparative Phase II trials.

## Background

Screening of potential anticancer agents in Phase II trials is a crucial step in the clinical development pathway. To increase efficiency and reduce costs, it is important to rapidly identify the most promising and efficacious agents to take forward into larger trials.

Several Phase II designs are available, including both single-arm and randomised approaches. Traditional methods such as Simon’s two-stage design [1] have been widely used in oncology [2]. However adaptive multi-stage Bayesian designs [3], with the opportunity to modify the trial at interim timepoints—for example, by stopping or expanding recruitment—are gaining popularity.

There is further scope to increase efficiency when researchers wish to evaluate several potential therapies concurrently. Possible options include running multiple single-arm trials successively or using randomised designs. Often in Phase II oncology trials, randomised designs are still non-comparative. That is, each arm is compared to a historical response rate rather than compared to another arm in the trial. There has been a substantial number of papers on adaptive multi-arm trials where there is a shared control group (see e.g., [4–6]), and ones without a control group [7–10].

Where adaptive designs are employed, delay in interim analyses (due to the time taken to follow-up patient outcomes and actually conduct the interim analysis) either reduces the efficiency provided by the adaptive design, or delays the progress of the trial [11]. The former occurs when recruitment is not paused at the interim analysis, and the latter when recruitment is paused whilst the interim analysis is ongoing. In the former case, there will be a group of patients who neither contribute information to the interim analysis nor benefit from the decision made [12]. The size of this group depends on the length of time the endpoint takes to observe and the recruitment rate. In an extreme scenario, if all patients are recruited before any patient has endpoint information measured, then the adaptive design provides no benefit to any patients in the trial. If the recruitment is paused whilst interim analyses are conducted then the trial duration will be prolonged by a period, depending on the length of time taken to evaluate these endpoints.

When there are multiple arms in a non-comparative Phase II trial, additional options are available for recruiting patients to different arms. Here, we discuss the Multi-Arm Sequential Trial with Efficient Recruitment (MASTER) design, which can provide an efficient and rapid assessment of multiple potential therapies within one protocol. This is undertaken by prioritising recruitment of participants to one arm at a time, with the priority arm changing while interim analyses are undertaken. This is similar to the ‘cassette’ approach employed in the Intergroup LEAP trial [13] and has been employed in WIRE, a Phase II, a window-of-opportunity trial evaluating a series of therapeutic agents in renal cell cancer (https://clinicaltrials.gov/ct2/show/NCT03741426) which will be used here as an exemplar.

In this paper, we evaluate how this approach compares to other potential designs in terms of the time taken to evaluate a number of treatment arms. We investigate how the time taken depends on changes in endpoint length and recruitment rate.

## Methods

### WIRE

WIRE (NCT03741426) is a non-randomised trial testing five monotherapy and combination therapy arms in the window-of-opportunity prior to surgery, usually around 4 weeks. Patients with surgically resectable renal cell cancer are treated with cediranib, cediranib and olaparib, olaparib, durvalumab or durvalumab and olaparib in the period prior to surgery. The primary outcome is biological evidence of treatment response (hereafter referred to as ‘biological response’), defined as a 30% increase in CD8-positive T cells for durvalumab containing arms and a 30% decrease in tumour capillary permeability measured using k^trans^ on DCE-MRI for all other arms. Each arm is tested in a three-stage single-arm trial with potential stopping for lack of efficacy or efficacy after each stage.

At each interim timepoint a Bayesian analysis is conducted to determine:

1. The posterior probability that the biological response rate is greater than 0.2 for monotherapy arms or 0.3 for combination therapy arms.
2. The predictive probability that the treatment would be found to be successful if the full enrolment was reached.

If the first probability is above 98% the arm is stopped for efficacy. If the second probability is below 2% the arm is stopped for lack of efficacy. If neither is true recruitment to the next stage will start. Assuming the number of participants per stage is as planned, these stopping rules can be translated into thresholds based on the observed number of biological responses at each interim analysis. Table 1 shows the stopping rules that are used for combination arms in the WIRE trial. Monotherapy arms are evaluated in a similar way but with different sample sizes and stopping rules.

**Table 1:** Stopping rules for the three-stage design.

Stage	Rule for efficacy stopping	Rule for futility stopping	Rule for continuing to next stage
1 (10 participants)	>6 responders	<=2 responders	3–6 responders
2 (15 participants)	>8 responders	<=5 responders	6–8 responders
3 (20 participants)	>9 responders	<=9 responders	NA

### Design comparison

**Fig. 1:**
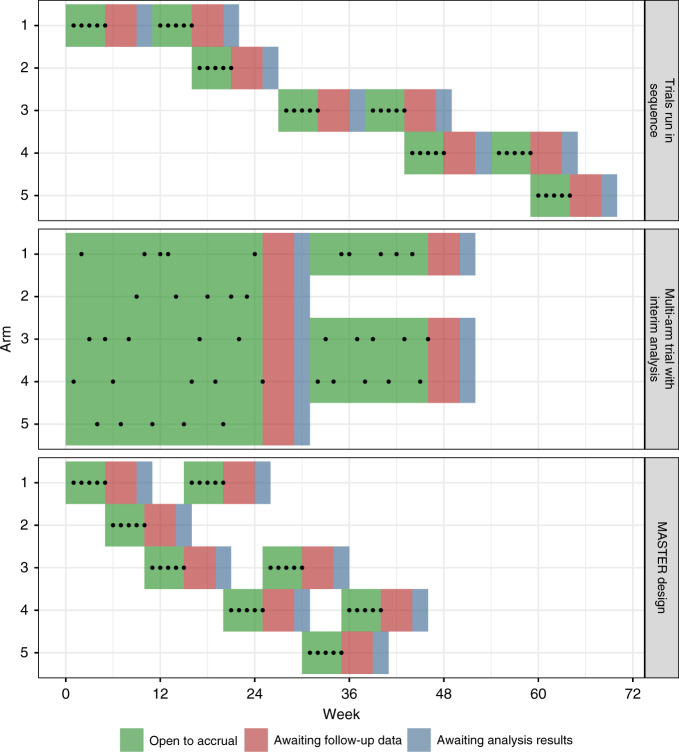
Illustration of the three different approaches, dots represent enrolled participants; green periods represent when the arm is open, red when the endpoint is being followed-up and blue the time it takes to conduct the interim analysis. For the colour figure, please see the online version.

We compare three different designs (illustrated in Fig. 1) for screening multiple new oncology drugs. For most simulation scenarios, we assume a maximum of 20 patients are recruited to each arm over three stages (ten, five and five patients in stages 1, 2 and 3, respectively). After stage 1, and stage 2 (if relevant), an interim analysis determines whether the trial should continue to the next stage by comparing the number of biological responses against pre-specified stopping rules given in Table 1. In two simulation scenarios, we vary the number of stages, as described later.

We assume *P* represents the probability of biological response for each participant, and the null hypothesis is H_0_: *P* ≤ 0.3 with the alternative hypothesis H_1_: *P* > 0.3. This design then provides ~93% power (with a 10% type-I error rate) to find significant efficacy when *P* = 0.6. Each design has the same type-I error rate and power for each arm. The difference is solely in terms of how patients are allocated.

The three different designs (Fig. 1, under the simplifying assumption that all stages consist of five patients) are as follows:

#### (1) Trials run in sequence

Each drug is tested one-by-one, in sequence. During each interim analysis, recruitment is paused. If the stopping rules are not met, recruitment is restarted and the trial continues. If stopping rules are met, the next arm is commenced. Once the last patient in the third stage of the trial is recruited, the next trial is opened for recruitment. This process continues until the last trial either stops at an interim analysis or completes stage 3.

#### (2) Multi-arm trial with interim analysis

All drugs are tested in parallel within a multi-arm trial. At each interim analysis timepoint, recruitment is paused until the analysis is complete. Any arms meeting the futility or efficacy stopping rule are dropped and recruitment resumes with the remaining arms. The trial continues until stage 3 is complete or all arms are stopped for futility or efficacy.

#### (3) MASTER design

The design prioritises arms in the order 1–5 (with arm 1 being the highest priority and arm 5 the lowest). The trial starts with arm 1 open for recruitment. All patients are allocated to arm 1 until it reaches the first stage sample size, at which point the interim analysis for arm 1 starts and arm 2 opens for recruitment. When each arm starts an interim or final analysis, recruitment is reopened to the highest priority arm that has not yet reached its total sample size and is not in an interim analysis. If all arms are complete or in an interim analysis, then recruitment is paused until an arm is reopened.

### Simulation setup

To evaluate the time taken by the three design approaches, we conducted a simulation study. This is summarised in the ADEMP framework proposed by Morris et al. [14] in Supplementary Table 1.

In each case, we assumed that participants became available for enrolment at a random set of times following an exponential distribution. The mean of this distribution was set so that the mean recruitment time per month was equal to a specified level (which was varied for the first simulation scenario and set to 4.5 per month for the second simulation scenario). As WIRE was a window-of-opportunity trial, we assume that a participant was only recruited to the trial if an arm was open at that time (i.e., enrolment could not be delayed beyond the simulated time).

After simulating the enrolment availability times for participants, we allocated them to an arm depending on the design. For the ‘Trials run in sequence’ and MASTER cases, there was only ever one arm open; for the multi-arm trial, the participant is allocated to the next arm in the sequence. We note that in practice, patients could be randomised between arms, for example using block randomisation, in a multi-arm trial design.

For each participant, the endpoint availability time was set to the enrolment time plus a specified endpoint assessment delay (which was set to 3 months for the first simulation study and varied for the second simulation study). This then determined when an arm was open to the interim analysis. A responder outcome was simulated for each participant following a Bernoulli distribution with the specified value of *P*.

It was assumed that half a month was required to conduct the interim analysis. The interim analysis would compare the number of responders observed in an arm to the stopping rules in Table 1. It was assumed that the trial design would always follow the stopping rules.

Each simulation replicate simulated a trial unfolding with patients becoming available for enrolment, allocated to arms according to the design, and arms undergoing interim analysis. The time of the last analysis completing was recorded, and this was summarised over 10,000 replicates.

We considered several possible values of *P*, the response probability. A ‘null’ scenario set the value of *P* for all arms to 0.3, an ‘intermediate’ scenario set the value to 0.45 and an ‘alternative’ scenario set the value to 0.6. Supplementary material shows results for a scenario where two arms had *P* = 0.3, one had *P* = 0.45 and two had *P* = 0.6.

We also investigated how two other variables affected the results. First, the number of arms: the null scenario above was repeated for trials of three arms and seven arms. Second the number of stages: the null scenario was repeated with two stages and four stages. For the two-stage design, the sample sizes at each stage were 10 and 10, with stopping at stage 1 if < =2 responses or >6 responses were observed. For the four-stage design, the sample size was 5 for each stage. The trial would stop after: stage 1 if there was < =1 responder or 5 responders; stage 2 if there were < =2 responders or >6 responders; and stage 3 if there were < =5 responders or >8 responders.

## Results

**Fig. 2:**
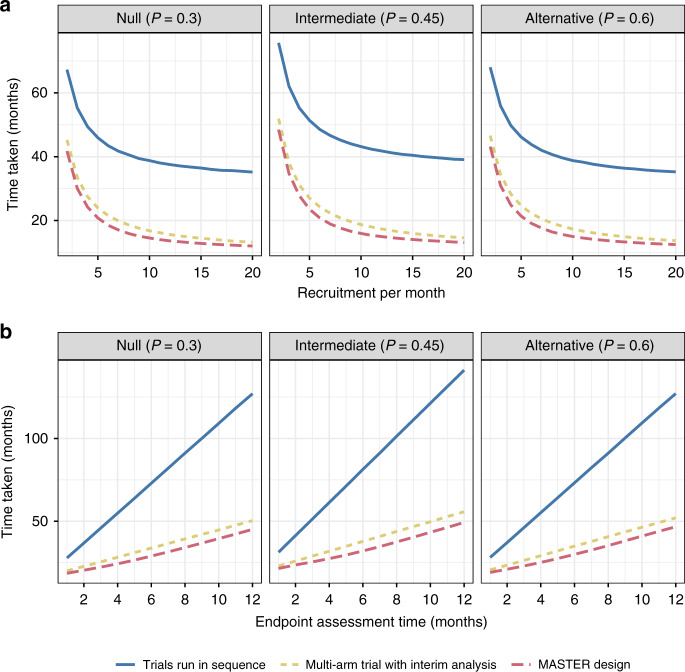
Comparison of time taken for scenarios when all arms are inefficacious (*P* = 0.3), intermediate (*P* = 0.45) and efficacious (*P* = 0.6). Panel **a** shows how this changes as recruitment per month changes (with endpoint length 3 months and interim analysis assumed to take half a month); panel **b** shows how this changes as endpoint length changes (assuming an average of 4.5 patients recruited per month).

We present the average time taken by each design to evaluate five treatment arms for three different scenarios (Fig. 2). The first scenario, the null scenario, assumes that all treatments are non-efficacious, with a biological response probability 0.3. The second scenario, the alternative scenario, assumes that all treatments are efficacious, with a biological response probability 0.6. The third scenario, the intermediate scenario, assumes all have an intermediate biological response probability of 0.45. Panel a shows the average time taken to evaluate the five treatments as the recruitment rate increases (assuming the endpoint takes 3 months to observe) and panel b shows the time taken as the endpoint length increases (assuming 4.5 patients per month recruited on average).

Running trials in sequence, even with an adaptive design, takes a considerable amount of additional time regardless of the recruitment rate or endpoint length. The multi-arm and MASTER design can considerably reduce the average time taken.

The average time taken is always the shortest in the MASTER design. Using realistic settings, it can reduce the time taken to evaluate five arms by 3–4 months. This advantage is maintained as the recruitment rate increases and endpoint delay increases. The greatest advantage appears to be for moderate-to-high recruitment rates in the intermediate scenario. This makes intuitive sense as the intermediate scenario is the one where arms are least likely to stop early. As a result, there are, on average, more analyses.

Supplementary Fig. 1 shows results for a scenario where treatments had different effects, showing a similar pattern to results in Fig. 2.

**Fig. 3:**
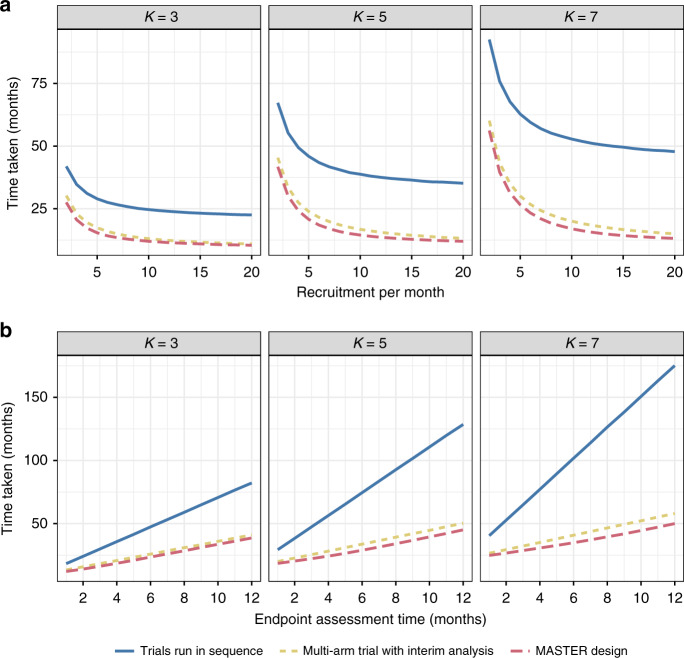
Comparison of time taken as the number of arms varies (three, five and seven) when all arms are inefficacious (*P* = 0.3). Panel **a** shows how this changes as recruitment per month changes (with endpoint length 3 months and interim analysis assumed to take half a month); panel **b** shows how this changes as endpoint length changes (assuming an average of 4.5 patients recruited per month).

We next considered the effect of varying the number of treatment arms (whilst keeping the number of stages at three). Figure 3 shows results for three arms, five arms and seven arms. As the number of arms increases, the trial will take longer on average. The relative advantage provided by MASTER remains similar, with a slight increase as the number of arms increases.

**Fig. 4:**
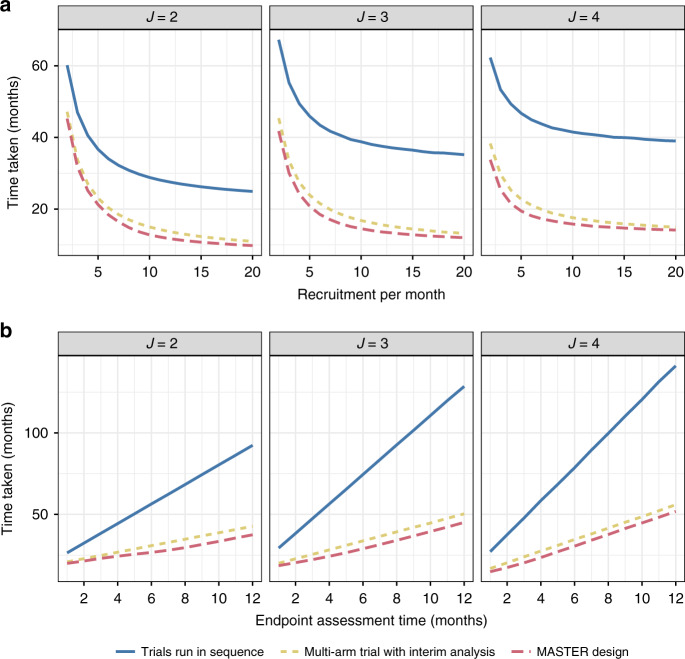
Comparison of time taken as the number of stages varies (two, three and four) when all arms are inefficacious (*P* = 0.3). Panel **a** shows how this changes as recruitment per month changes (with endpoint length 3 months and interim analysis assumed to take half a month); panel **b** shows how this changes as endpoint length changes (assuming an average of 4.5 patients recruited per month).

Last, we considered the effect of varying the number of stages. As described in the methods, we considered two stages, three stages and four stages. Figure 4 shows the results of the comparisons. Again, the advantage of the MASTER design is maintained. Interestingly, when there is a delay, additional interim analyses do not necessarily reduce the average time taken by the trial.

## Discussion

With the need to efficiently test multiple oncology treatments in a time- and cost-effective manner, trial designs that can contribute are a priority. Here, we have evaluated how a design that can flexibly close and open recruitment to arms improves the time taken, as shown by the WIRE trial exemplar. A recent review of the use of adaptive non-comparative oncology trials [2] identified several trials that might have benefited from the proposed approach [15–18], as well as the WIRE trial and LEAP trial [13].

We focused on the setting where recruitment is paused whilst interim analyses are conducted, as in a previous adaptive RCC trial of imaging modalities [19] and in WIRE. In practice, recruitment may be continued whilst awaiting follow-up and interim analysis. We note that the advantage of the MASTER design would remain as it would minimise the ‘overrun’ of patients who are recruited unnecessarily to treatment arms that ended up being dropped at the interim analysis. A limitation of our simulation study is that we assume patients are available for recruitment for an instant of time and lost to the trial if no arms are available at that instant. However, depending on the clinical setting, a potential patient may be able to wait for an arm to be open, which would reduce the effect of this delay. As the WIRE trial is a window-of-opportunity trial, this period is likely to be very short, so the results are not too sensitive to this assumption.

The MASTER design also has the advantage that it will generate completed results on arms as it proceeds. If the trial has a fixed duration, it may be preferable to collect complete data on a subset of arms rather than incomplete data on all. The arms may be ordered in priority in this case. When some arms are monotherapies and others are combinations, the MASTER design allows additional safety data to be collected on monotherapies prior to enrolling patients on combinations. It may also allow information from earlier cohorts (e.g. from translational analyses) to modify the design of subsequent cohorts. Lastly, the MASTER design minimises the amount of time the trial is closed to recruitment; this helps overcome the practical issues resulting from closing and open recruitment.

A disadvantage of the MASTER design may occur when there are multiple pharmaceutical companies involved in the trial. A company may be reluctant to support a trial in which their product is lower down in the order of priority. This is not an issue for WIRE, where all arms are provided by a single company. If the recruitment rate allows, a hybrid design could randomly allocate patients between sub-trials, each one testing a particular portfolio for each pharmaceutical company. Each individual sub-trial could then utilise the MASTER design.

The parallel multi-arm trial may have other advantages if patients are randomly allocated. This may reduce the risk of selection bias and ensure arms are likely to be comparable in terms of the patient population, allowing better comparison between arms. In our experience, selection bias is a low risk in practice as the main priority of investigators is recruiting all eligible patients. It is possible to consider an approach that mixes elements of the parallel multi-arm trial and the MASTER design, e.g., through gradually closing arms prior to an interim analysis and gradually opening them as interim results are available. In the case of slow recruitment, this may make the parallel multi-arm design more robust to delay in a similar manner to MASTER.

An interesting result that arose from varying the number of stages was that the average time taken did not necessarily decrease as the number of interims increased. This goes against published work in the methodological literature that generally shows more interims provides more efficiency gain (albeit with diminishing returns). Further investigation of this finding for a broader set of adaptive designs would be of interest. We would recommend that designers of clinical trials consider the delay when comparing different designs. In this paper, we based stopping rules on the WIRE trial that were informed from Bayesian approaches; the results and relative performance of the designs may vary if different thresholds are used.

The MASTER design could be appropriate to apply in settings other than the one considered here. First, its advantage does not depend on the endpoint used, so could be used when a continuous outcome [20] or (with some more complexity) a time-to-event outcome is used. Second, it could be applied in a randomised platform trial with a shared control group. This may provide efficiency when there is a delay in the endpoint. However, since the efficiency advantage of the shared control group increases as the number of experimental arms in the trial at the same time increases, we are unsure that the MASTER design would have a similar efficiency advantage as demonstrated here.

In conclusion, we believe the MASTER design, employed in WIRE, is a promising method to improve the efficiency of early-phase oncology trials.

## Supplementary information

Supplementary material

## Data Availability

Simulation study code to reproduce the results is provided as supplementary material.
