# Supplementary material for: Advantages of multi-arm non-randomised sequentially allocated cohort designs for Phase II oncology trials
Source: Br J Cancer. 2021 Nov 8;126(2):204–10. doi: 10.1038/s41416-021-01613-5 (PMC8770479; doi:10.1038/s41416-021-01613-5)
Supplement: Supplementary file 1 — Supplementary material [file 41416_2021_1613_MOESM1_ESM.docx]

Supplementary material for “Advantages of multi-arm non-randomized sequentially allocated cohort designs for phase II oncology trials”

Supplementary Table 1 - ADEMP framework for simulation study

| Aims | To compare three approaches to conducting multiple single-arm trials when there is delay in the outcome. |
| --- | --- |
| Data-generating mechanism | Simulated recruitment times assuming Poisson arrival time, simulated outcomes using the Bernoulli distribution.  Bernoulli probabilities are varied across simulation scenarios. Within each scenario, the mean of the Poisson arrival time is varied to match an average recruitment rate. |
| Estimand | Time taken by trial to complete evaluation of all arms. |
| Methods | - Trials run in sequence  - Parallel multi-arm design  - MASTER design  All are described in Methods section of main paper. |
| Performance measures | Mean time taken by trial to complete, which is a good measure of the efficiency of the design. |

**Supplementary Figure 1**

Comparison of time taken in the scenario when arms 1 and 2 had p=0.3, arm 3 had p=0.45, and arms 4 and 5 had p=0.6. Panel A shows how this changes as recruitment per month changes (with endpoint length 3 months and interim analysis assumed to take half a month); panel B shows how this changes as endpoint length changes (assuming average of 4.5 patients recruited per month)

**A**

**
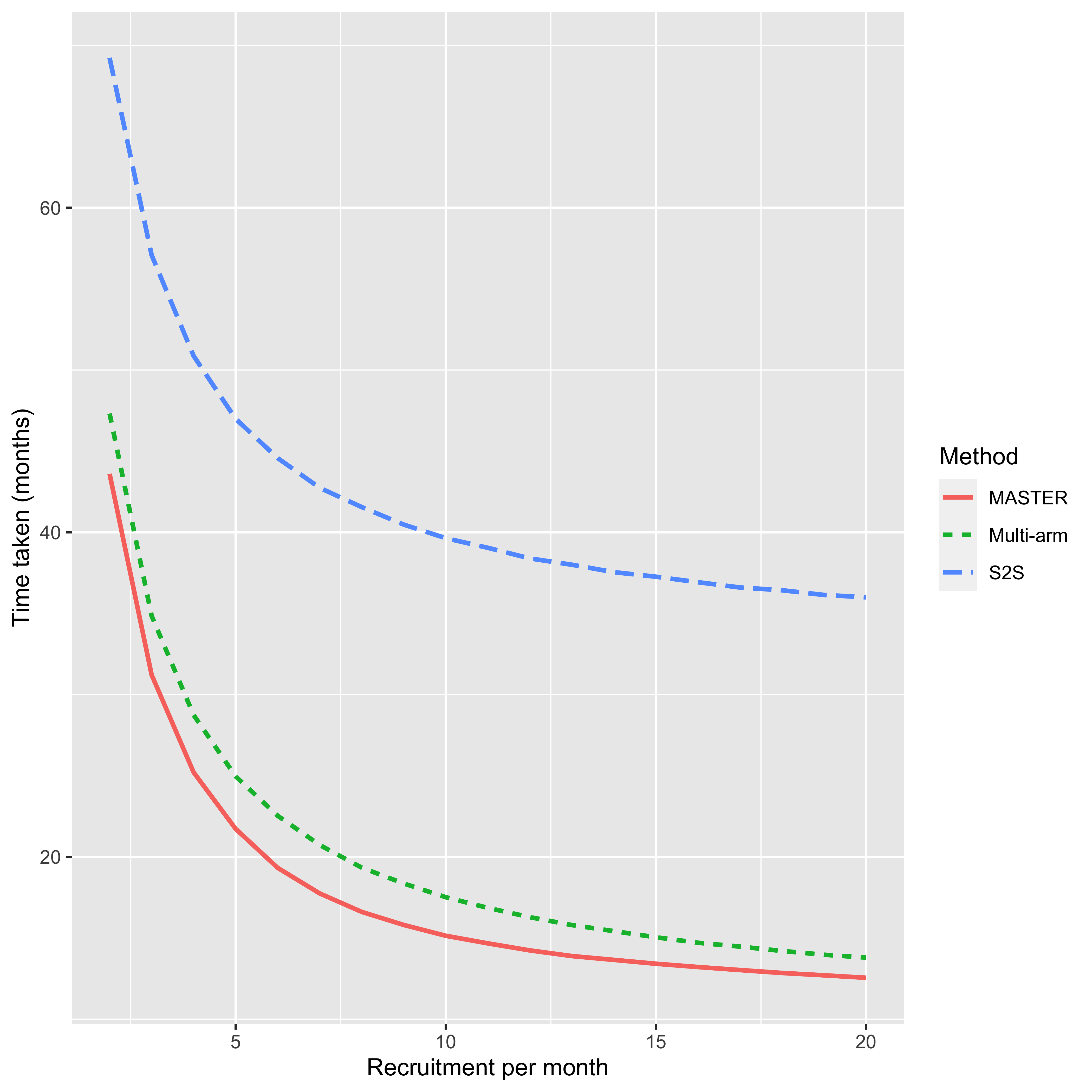
**

**B**

**
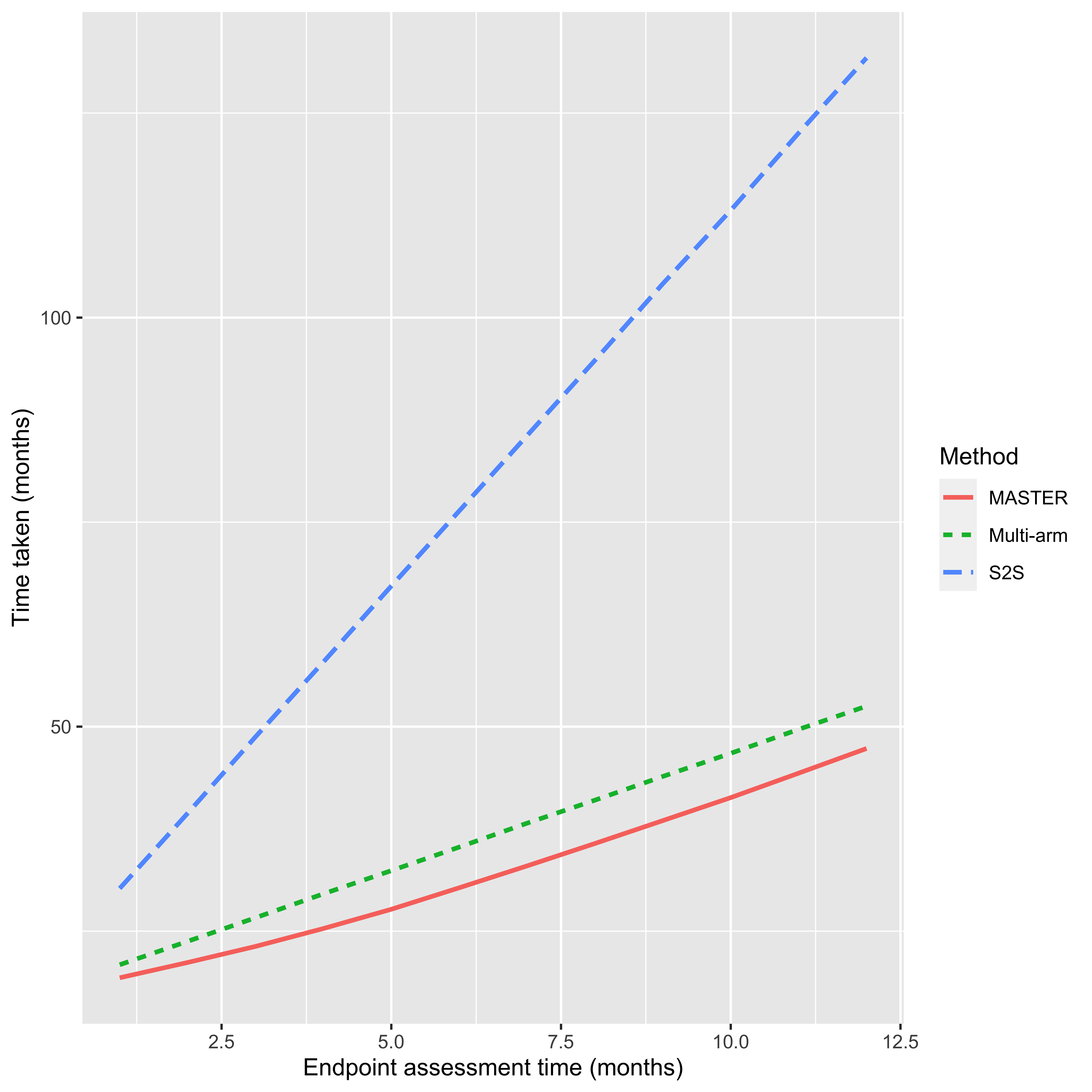
**

**R code for simulations**

#Simulation code for WIRE design paper

#Input parameters:

number.arms=5 #number of arms

response.prob=c(0.6,0.6,0.6,0.6,0.6) #Vector of length number.arms with probability of response

endpoint.length=3 #number of time units to observe an individual's response

interim.analysis.time=0.5 #Number of time units to conduct and implement results of an interim analysis

recruitment.per.timeunit=5 #Number of patients recruited per time unit

#Parameters used in WIRE:

#Number of patients to be allocated in each stage

n.perstage=c(10,5,5)

#futility.responses: if number of responders is equal to or lower than this, then stop for futility

futility.responses=c(2,5,9)

#efficacy.responses: if number of responders is higher than this, then stop for efficacy

efficacy.responses=c(6,8,9)

#Simulate WIRE-type design that prioritises the lower numbered cohorts and solely recruits to one cohort at a time

#n.perstage: vector with number of stages - number of patients recruited in each stage in each cohort

#futility.responses: vector with number of entries equal to number of stages - if at the relevant stage, number of responders is equal to or lower than this, then stop for futility

#efficacy.responses: vector with number of stages equal to number of stages - if at the relevant stage number of responders is higher than this, then stop for efficacy

#interim.analysis.time: number of time units required to conduct interim analysis

#endpoint.length: time taken for patient's response to be ascertained

#recruitment.per.timeunit: number of patients recruited per time unit

master.properties=function(number.arms=5,n.perstage=c(10,5,5),futility.responses=c(2,5,9),efficacy.responses=c(6,8,9),interim.analysis.time=0.5,endpoint.length=3,recruitment.per.timeunit=5)

{

#Assume max patients is maximum number of patients needed to be enrolled x10 to allow for periods where trial might be closed

maxpatients=(sum(n.perstage)*number.arms)*10

patientid=1:maxpatients

#cumulative sample size at each stage:

cumulative.n.perstage=cumsum(n.perstage)

#deterministic recruitment: recruitmenttime=seq(0,maxpatients/recruitment.per.timeunit,length.out=maxpatients)

recruitmenttime=cumsum(rexp(maxpatients,rate=recruitment.per.timeunit))

assessmenttime=recruitmenttime+endpoint.length

#cycle through patients and determine which cohort they are included in

cohortopen=rep(1,number.arms)

allocation=rep(0,maxpatients)

npercohort=rep(0,number.arms)

reopentime=rep(Inf,number.arms)

cohortcomplete=rep(0,number.arms)

response=rep(NA,maxpatients)

futilitystop=rep(0,number.arms)

efficacystop=rep(0,number.arms)

rejecth0=rep(0,number.arms)

#keeps track of which stage each cohort is in

whichstage=rep(1,number.arms)

patient=0

while(patient < maxpatients & sum(cohortcomplete)<number.arms)

{

patient=patient+1

#determine which arms are open:

arm.toallocate=suppressWarnings(min(which(cohortopen==1 & cohortcomplete==0)))

if(is.infinite(arm.toallocate))

{

allocation[patient]=arm.toallocate

for(k in 1:number.arms)

{

if(reopentime[k]<recruitmenttime[patient] & cohortcomplete[k]==0)

{

cohortopen[k]=1

}

}

next;

}

#otherwise, set allocation of next set of patients:

patientids.toallocate=patient:(patient+n.perstage[whichstage[arm.toallocate]]-1)

allocation[patientids.toallocate]=arm.toallocate

response[patientids.toallocate]=rbinom(length(patientids.toallocate),1,response.prob[arm.toallocate])

npercohort[arm.toallocate]=npercohort[arm.toallocate]+length(patientids.toallocate)

#conduct analysis for this cohort:

cohortopen[arm.toallocate]=0

#Code to do interim/final analysis:

numberresponses=sum(response[allocation==arm.toallocate])

if(numberresponses<=futility.responses[whichstage[arm.toallocate]])

{

futilitystop[arm.toallocate]=1

rejecth0[arm.toallocate]=0

cohortcomplete[arm.toallocate]=1

}

if(numberresponses>efficacy.responses[whichstage[arm.toallocate]])

{

efficacystop[arm.toallocate]=1

rejecth0[arm.toallocate]=1

cohortcomplete[arm.toallocate]=1

}

#otherwise continue recruitment to next stage

whichstage[arm.toallocate]=whichstage[arm.toallocate]+1

#If interim analysis leads to continuing enrollment, then calculate minimum reopening time

#Minimum time that cohort can be reopened, recruitment time of current patient: assessment time of current patient + time taken to conduct interim analysis

reopentime[arm.toallocate]=assessmenttime[patientids.toallocate[length(patientids.toallocate)]]+interim.analysis.time

#set patient to final patientids.toallocate

patient=patientids.toallocate[length(patientids.toallocate)]

#determine if any cohorts are to reopen

for(k in 1:number.arms)

{

if(reopentime[k]<recruitmenttime[patient+1] & cohortcomplete[k]==0)

{

cohortopen[k]=1

}

}

}

response=response[-which(allocation==0)]

recruitmenttime=recruitmenttime[-which(allocation==0)]

assessmenttime=assessmenttime[-which(allocation==0)]

allocation=allocation[-which(allocation==0)]

numberpatients.noallocation=length(allocation[is.infinite(allocation)])

timetaken=assessmenttime[length(assessmenttime)]+interim.analysis.time

return(list(timetaken=timetaken,numberpatients.noallocation=numberpatients.noallocation,allocation=allocation))

}

#Calculate average properties of conducting Simon two stage designs in sequence (although start next trial whilst previous one is in follow-up for stage 2)

#n1.S2S: number of patients recruited in first stage in each cohort

#n2.S2S: number of patients recruited in second stage (if it occurs) in each cohort

#r1.S2S: number of responses needed in first stage to continue to second stage (if number responses strictly below, the cohort stops for futility)

#r.S2S: number of combined stage 1 and stage 2 responses required to declare promising treatment at end of stage 2 for a cohort

#interim.analysis.time: number of time units required to conduct interim analysis

#endpoint.length: time taken for patient's response to be ascertained

#recruitment.per.timeunit: number of patients recruited per time unit

S2S.properties=function(number.arms=5,n.perstage=c(10,5,5),futility.responses=c(2,5,9),efficacy.responses=c(6,8,9),interim.analysis.time=0.5,endpoint.length=3,recruitment.per.timeunit=5)

{

#sequential Simon two-stage designs time taken:

maxpatients=(sum(n.perstage)*number.arms)*100

patientid=1:maxpatients

#cumulative sample size at each stage:

cumulative.n.perstage=cumsum(n.perstage)

#deterministic recruitment: recruitmenttime=seq(0,maxpatients/recruitment.per.timeunit,length.out=maxpatients)

recruitmenttime=cumsum(rexp(maxpatients,rate=recruitment.per.timeunit))

assessmenttime=recruitmenttime+endpoint.length

#cycle through patients and determine which cohort they are included in

cohortopen=rep(1,number.arms)

allocation=rep(0,maxpatients)

npercohort=rep(0,number.arms)

reopentime=rep(Inf,number.arms)

cohortcomplete=rep(0,number.arms)

response=rep(NA,maxpatients)

futilitystop=rep(0,number.arms)

efficacystop=rep(0,number.arms)

rejecth0=rep(0,number.arms)

#keeps track of which stage each cohort is in

whichstage=rep(1,number.arms)

whicharms=1:number.arms

patientid=1

for(arm in 1:number.arms)

{

for(stage in 1:length(n.perstage))

{

patientids.toallocate=patientid:(patientid-1+n.perstage[stage])

allocation[patientids.toallocate]=rep(arm,n.perstage[stage])

response[patientids.toallocate]=rbinom(n.perstage[stage],1,response.prob[arm])

#do interim analysis:

numberresponses=sum(response[allocation==arm])

if(numberresponses<=futility.responses[stage])

{

futilitystop[arm]=1

rejecth0[arm]=0

cohortcomplete[arm]=1

}

if(numberresponses>efficacy.responses[stage])

{

efficacystop[arm]=1

rejecth0[arm]=1

cohortcomplete[arm]=1

}

#update patient id:

if(stage==length(n.perstage) & arm < number.arms)

{

patientid=patientids.toallocate[length(patientids.toallocate)]

allocation[(patientids.toallocate[length(patientids.toallocate)]+1):patientid]=Inf

}

if(stage<length(n.perstage) | arm == number.arms)

{

interimanalysisfinished.time=assessmenttime[patientids.toallocate[length(patientids.toallocate)]]+interim.analysis.time

patientid=min((1:maxpatients)[which(recruitmenttime>interimanalysisfinished.time)])

allocation[(patientids.toallocate[length(patientids.toallocate)]+1):patientid]=Inf

}

if(cohortcomplete[arm]==1)

{

break;

}

}

#if all arms are finished then stop

if(mean(cohortcomplete)==1)

{

break;

}

}

if(sum(ifelse(allocation==number.arms,1,0))==sum(n.perstage))

{

lastpatientid=max((1:length(allocation))[allocation==number.arms])

allocation=allocation[1:lastpatientid]

response=response[1:lastpatientid]

recruitmenttime=recruitmenttime[1:lastpatientid]

assessmenttime=assessmenttime[1:lastpatientid]

}

#check why following code is going wrong

response=response[which(allocation!=0)]

recruitmenttime=recruitmenttime[which(allocation!=0)]

assessmenttime=assessmenttime[which(allocation!=0)]

allocation=allocation[which(allocation!=0)]

#if trial gets to stage 3 analysis of final arm, then delete any inf allocation after that:

numberpatients.noallocation=length(allocation[is.infinite(allocation)])

timetaken=assessmenttime[length(assessmenttime)]+interim.analysis.time

return(list(timetaken=timetaken,numberpatients.noallocation=numberpatients.noallocation,allocation=allocation))

}

#Simulate trials in parallel with interim analyses after each set of recruitment

#n.perstage: vector with number of stages - number of patients recruited in each stage in each cohort

#futility.responses: vector with number of entries equal to number of stages - if at the relevant stage, number of responders is equal to or lower than this, then stop for futility

#efficacy.responses: vector with number of stages equal to number of stages - if at the relevant stage number of responders is higher than this, then stop for efficacy

#interim.analysis.time: number of time units required to conduct interim analysis

#endpoint.length: time taken for patient's response to be ascertained

#recruitment.per.timeunit: number of patients recruited per time unit

multiarm.properties=function(number.arms=5,n.perstage=c(10,5,5),futility.responses=c(2,5,9),efficacy.responses=c(6,8,9),interim.analysis.time=0.5,endpoint.length=3,recruitment.per.timeunit=5)

{

#Assume max patients is maximum number of patients needed to be enrolled x10 to allow for periods where trial might be closed

maxpatients=(sum(n.perstage)*number.arms)*10

patientid=1:maxpatients

#cumulative sample size at each stage:

cumulative.n.perstage=cumsum(n.perstage)

#deterministic recruitment: recruitmenttime=seq(0,maxpatients/recruitment.per.timeunit,length.out=maxpatients)

recruitmenttime=cumsum(rexp(maxpatients,rate=recruitment.per.timeunit))

assessmenttime=recruitmenttime+endpoint.length

#cycle through patients and determine which cohort they are included in

cohortopen=rep(1,number.arms)

allocation=rep(0,maxpatients)

npercohort=rep(0,number.arms)

reopentime=rep(Inf,number.arms)

cohortcomplete=rep(0,number.arms)

response=rep(NA,maxpatients)

futilitystop=rep(0,number.arms)

efficacystop=rep(0,number.arms)

rejecth0=rep(0,number.arms)

#keeps track of which stage each cohort is in

whichstage=rep(1,number.arms)

whicharms=1:number.arms

patientid=1

for(stage in 1:length(n.perstage))

{

patientids.toallocate=patientid:(patientid-1+n.perstage[stage]*length(whicharms))

allocation[patientids.toallocate]=rep(whicharms,n.perstage[stage])

response[patientids.toallocate]=rbinom(n.perstage[stage]*length(whicharms),1,response.prob[allocation[patientids.toallocate]])

#do interim analysis:

for(arm in whicharms)

{

numberresponses=sum(response[allocation==arm])

if(numberresponses<=futility.responses[stage])

{

futilitystop[arm]=1

rejecth0[arm]=0

cohortcomplete[arm]=1

}

if(numberresponses>efficacy.responses[stage])

{

efficacystop[arm]=1

rejecth0[arm]=1

cohortcomplete[arm]=1

}

}

#if all arms are finished then stop

if(mean(cohortcomplete)==1)

{

break;

}

#update patient id:

interimanalysisfinished.time=assessmenttime[patientids.toallocate[length(patientids.toallocate)]]+interim.analysis.time

patientid=min((1:maxpatients)[which(recruitmenttime>interimanalysisfinished.time)])

allocation[(patientids.toallocate[length(patientids.toallocate)]+1):patientid]=Inf

whicharms=(1:number.arms)[cohortcomplete==0]

}

response=response[-which(allocation==0)]

recruitmenttime=recruitmenttime[-which(allocation==0)]

assessmenttime=assessmenttime[-which(allocation==0)]

allocation=allocation[-which(allocation==0)]

numberpatients.noallocation=length(allocation[is.infinite(allocation)])

timetaken=assessmenttime[length(assessmenttime)]+interim.analysis.time

return(list(timetaken=timetaken,numberpatients.noallocation=numberpatients.noallocation,allocation=allocation))

}

recruitmentresults=data.frame(Method=NULL,Recruitment.rate=NULL,Time.taken=NULL,Average.patients.missed=NULL)

for(recruitment.per.timeunit in 2:20)

{

print(recruitment.per.timeunit)

results.multiarm=replicate(multiarm.properties(recruitment.per.timeunit=recruitment.per.timeunit),n=10000)

averagetimetaken.multiarm=mean(as.double(results.multiarm[1,]))

averagenumberpatientsmissed.multiarm=mean(as.double(results.multiarm[2,]))

recruitmentresults=rbind(recruitmentresults,data.frame(Method="Multi-arm",Recruitment.rate=recruitment.per.timeunit,Time.taken=averagetimetaken.multiarm,Average.patients.missed=averagenumberpatientsmissed.multiarm))

set.seed(recruitment.per.timeunit)

results.master=replicate(master.properties(recruitment.per.timeunit=recruitment.per.timeunit),n=10000)

averagetimetaken.master=mean(as.double(results.master[1,]))

averagenumberpatientsmissed.master=mean(as.double(results.master[2,]))

recruitmentresults=rbind(recruitmentresults,data.frame(Method="MASTER",Recruitment.rate=recruitment.per.timeunit,Time.taken=averagetimetaken.master,Average.patients.missed=averagenumberpatientsmissed.master))

results.S2S=replicate(S2S.properties(recruitment.per.timeunit=recruitment.per.timeunit),n=10000)

averagetimetaken.S2S=mean(as.double(results.S2S[1,]))

averagenumberpatientsmissed.S2S=mean(as.double(results.S2S[2,]))

recruitmentresults=rbind(recruitmentresults,data.frame(Method="S2S",Recruitment.rate=recruitment.per.timeunit,Time.taken=averagetimetaken.S2S,Average.patients.missed=averagenumberpatientsmissed.S2S))

}

#plot results

library(ggplot2)

plot.recruitment=ggplot(recruitmentresults,aes(x=Recruitment.rate,y=Time.taken,group=Method,color=Method,linetype=Method))+geom_line(size=1)+scale_x_continuous(name = "Recruitment per month")+scale_y_continuous(name="Time taken (months)")

ggsave("Varyingrecruitmentrate.png",plot = plot.recruitment,dpi=600)

#Repeat with changing the endpoints length with recruitment per time unit of 4.5

endpointresults=data.frame(Method=NULL,Endpointlength=NULL,Time.taken=NULL,Average.patients.missed=NULL)

for(endpoint.length in 1:12)

{

print(endpoint.length)

results.multiarm=replicate(multiarm.properties(endpoint.length = endpoint.length ,recruitment.per.timeunit=4.5),n=10000)

averagetimetaken.multiarm=mean(as.double(results.multiarm[1,]))

averagenumberpatientsmissed.multiarm=mean(as.double(results.multiarm[2,]))

endpointresults=rbind(endpointresults,data.frame(Method="Multi-arm",Endpointlength=endpoint.length,Time.taken=averagetimetaken.multiarm,Average.patients.missed=averagenumberpatientsmissed.multiarm))

results.master=replicate(master.properties(endpoint.length = endpoint.length ,recruitment.per.timeunit=4.5),n=10000)

averagetimetaken.master=mean(as.double(results.master[1,]))

averagenumberpatientsmissed.master=mean(as.double(results.master[2,]))

endpointresults=rbind(endpointresults,data.frame(Method="MASTER",Endpointlength=endpoint.length,Time.taken=averagetimetaken.master,Average.patients.missed=averagenumberpatientsmissed.master))

results.S2S=replicate(S2S.properties(endpoint.length = endpoint.length),n=10000)

averagetimetaken.S2S=mean(as.double(results.S2S[1,]))

averagenumberpatientsmissed.S2S=mean(as.double(results.S2S[2,]))

endpointresults=rbind(endpointresults,data.frame(Method="S2S",Endpointlength=endpoint.length,Time.taken=averagetimetaken.S2S,Average.patients.missed=averagenumberpatientsmissed.S2S))

}

plot.endpoint=ggplot(endpointresults,aes(x=Endpointlength,y=Time.taken,group=Method,color=Method,linetype=Method))+geom_line(size=1)+scale_x_continuous(name = "Endpoint assessment time (months)")+scale_y_continuous(name="Time taken (months)")

ggsave("Varyingendpointlength.png",plot = plot.endpoint,dpi=600)

endpointresults.alt=endpointresults

recruitmentresults.alt=recruitmentresults
